# On the influence of several factors on pathway enrichment analysis

**DOI:** 10.1093/bib/bbac143

**Published:** 2022-04-23

**Authors:** Sarah Mubeen, Alpha Tom Kodamullil, Martin Hofmann-Apitius, Daniel Domingo-Fernández

**Affiliations:** 1 Department of Bioinformatics, Fraunhofer Institute for Algorithms and Scientific Computing, Sankt Augustin 53757, Germany; 2 Bonn-Aachen International Center for Information Technology (B-IT), University of Bonn, 53115 Bonn, Germany; 3 Fraunhofer Center for Machine Learning, Germany; 4 Enveda Biosciences, Boulder, CO, 80301, USA

**Keywords:** pathway enrichment, gene set analysis, pathway database, omics data, benchmark, gene set collection

## Abstract

Pathway enrichment analysis has become a widely used knowledge-based approach for the interpretation of biomedical data. Its popularity has led to an explosion of both enrichment methods and pathway databases. While the elegance of pathway enrichment lies in its simplicity, multiple factors can impact the results of such an analysis, which may not be accounted for. Researchers may fail to give influential aspects their due, resorting instead to popular methods and gene set collections, or default settings. Despite ongoing efforts to establish set guidelines, meaningful results are still hampered by a lack of consensus or gold standards around how enrichment analysis should be conducted. Nonetheless, such concerns have prompted a series of benchmark studies specifically focused on evaluating the influence of various factors on pathway enrichment results. In this review, we organize and summarize the findings of these benchmarks to provide a comprehensive overview on the influence of these factors. Our work covers a broad spectrum of factors, spanning from methodological assumptions to those related to prior biological knowledge, such as pathway definitions and database choice. In doing so, we aim to shed light on how these aspects can lead to insignificant, uninteresting or even contradictory results. Finally, we conclude the review by proposing future benchmarks as well as solutions to overcome some of the challenges, which originate from the outlined factors.

## Introduction

Pathway enrichment analysis has become one of the foremost methods for the interpretation of biological data as it facilitates the reduction of high-dimensional information to just a handful of biological processes underlying specific phenotypes. Over the last decade, the popularity of pathway enrichment analysis has led to the development of numerous different methods that can be categorized into three generations: (i) over-representation analysis (ORA), (ii) functional class scoring (FCS) and (ii) pathway topology (PT)-based, each of which adds an increasing layer of complexity to the analysis [1]. ORA, the first of the three, refers to a class of methods designed to identify gene sets that share a larger number of genes in common with a list of differentially expressed genes (DEGs) than would be expected by chance. Given a list of DEGs, a gene set and their complements, a statistical test is conducted to assess whether DEGs are over-represented in the gene set. Though simple to conduct, ORA methods rely upon arbitrary, and at times harsh, cutoffs to determine what constitutes a DEG. To remedy this problem, FCS methods test whether genes of a gene set have coordinated activity with the phenotype under study by using metrics to assign differential expression scores to each gene in the experiment. Genes are then ranked by their scores, which are subsequently used to calculate gene set scores and determine gene sets that are interesting in some statistically significant way. Finally, PT-based approaches build upon the latter class of methods and are characterized as additionally taking PT information into account, rather than solely relying upon gene sets, which lack interaction information. Thus, a formal distinction can be made between gene sets and pathways. Specifically, a gene set refers to a set of unranked genes which can be variously grouped, such as by their membership within a biological pathway or chromosomal position, while a pathway refers to a set of genes as well as any pairwise interactions between them. While the simplicity and accessibility of enrichment methods have been the main drivers to their widespread adoption by the community, the broad pool of methods at hand and the lack of gold standards pose a challenge in evaluating the variability of enrichment results. Consequently, several guidelines have been published in recent years on recommendations for the experimental design of an enrichment analysis [2–4].

An analogous but more philosophical debate in the community pertains to the choice of pathway or gene set database. Its selection is arguably one of the most decisive factors influencing the results of enrichment analyses as it determines the possible gene sets that can be enriched (i.e. genes within a gene set are enriched in an examined list of genes). The number of public databases has continued to grow in the past years in parallel with novel enrichment methods. However, the list of the most widely used databases has not changed in the last decade as enrichment analyses are predominantly conducted exclusively on one of the following three databases: KEGG [5], Reactome [6] and Gene Ontology (GO) [7]. While this selected group of databases comes with several advantages (e.g. large coverage of biological processes and regular updates), definitions of what constitutes a given pathway or gene set may be arbitrarily drawn across databases.

At present, users are offered a wide spectrum of enrichment methods and databases when performing enrichment analyses. This poses a challenge when considering the numerous factors that play a role in results of enrichment analysis, which can lead to insignificant, irrelevant or even contradictory results. Thus, in recent years, several benchmark studies have been conducted to evaluate the effects of various aspects of pathway analysis for practical guidelines.

**Figure 1:**
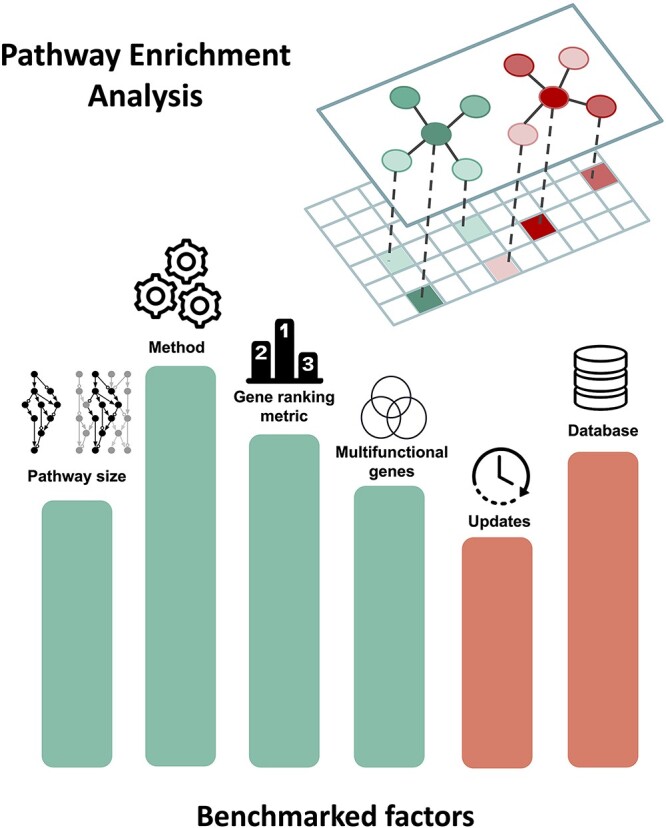
Illustration of major factors that influence the results of pathway enrichment analysis discussed in this review. The height and color of the bars are symbolic and do not correlate with importance. The two networks depicted above represent two biological pathways mapped to gene expression data (matrix below).

In this work, we review the findings of major benchmarks conducted on different factors that influence the results of pathway enrichment analysis (Figure 1). The goal of our paper is to both inform the broader community of researchers using pathway enrichment analysis of these factors and to summarize the findings of all the most recent benchmarks. Finally, we also discuss possible solutions to address these factors as well as other factors that have not yet been investigated but can be benchmarked in the future.

## Comparative studies on enrichment methods

Given the popularity of pathway enrichment analysis, at least 70 different methods have been developed as well as hundreds of variants [8, 9] (see Xie *et al*. [10] for an exhaustive survey of methods and benchmarks). The implementations of these methods can differ based on a number of factors, such as the gene-level statistic (e.g. *t*-test statistic and fold change), the gene set–level statistic (e.g. Kolmogorov–Smirnov (KS) statistic [11] and Wilcoxon rank sum test [12]), the formulation of the null and alternative hypotheses and the significance estimate. Many of the most commonly employed pathway enrichment methods have been compared in several major benchmarks and reviews. In this section, we outline the findings of 12 comprehensive comparative studies on enrichment methods (Table 1; for more details, see Supplementary Tables 1–3 available online at https://academic.oup.com/bib).

**Table 1:** Comparative studies evaluating differences across enrichment methods

No.	Review	Methods tested	Datasets	Database (# of gene sets/pathways)	Types of evaluated methods
1	[13]	7	36	KEGG (116)	Topology- and non-topology-based methods
2	[2]	10	75	KEGG (323) and GO (4631)	ORA and FCS methods
3	[3]	7	118	KEGG (232)	Topology-based methods
4	[14]	6	20	KEGG (86)	Topology- and non-topology-based methods
5	[15]	9	3	KEGG (114)	Topology-based methods
6	[16]	13	6	GO gene set collection extracted from MSigDB [17] v6.1 (5917)	Widely used pathway enrichment methods
7	[18]	8	3	MSigDB v5.0 (10,295)	Widely used pathway enrichment methods
8	[9]	10	86	KEGG; 150 pathways for all methods except 130 for PathNet [19] and 186 for CePa [20, 21]	Topology- and non-topology-based methods
9	[22]	11	1	C2 collection from MSigDB v4.0 (4722)	Methods differing based on null hypothesis
10	[23]	16	42	KEGG (259) and Metacore™ (88)	ORA and FCS methods
11	[24]	5	6	KEGG (192)	ORA and FCS methods
12	[25]	7	38	KEGG (189)	ORA and FCS methods

In the third column, we report the number of enrichment methods compared in each study (see Supplementary Tables 2 and 3, available online at https://academic.oup.com/bib, for details on the methods tested). Here, we would like to note that we differentiate between methods and tools/web applications based on Geistlinger *et al.* [2]. In the fourth column, we report the number of datasets each study performed comparisons on, all of which were experimental datasets except in [3, 13, 14, 18, 22], which included both experimental and simulated datasets. Finally, the fifth column reports the pathway databases used in each study while the number of pathways is shown between parentheses.

### Metrics for method evaluation

A particular challenge in the design of comparative studies on enrichment methods is that in the absence of a comprehensive understanding of the complex biological processes involved across experimental conditions, results are often not verifiable beyond retrospective evaluations. That is to say, without a gold standard with which to compare the results produced by any given method, conclusive assessments are often difficult to make. Nonetheless, several techniques to compare methods are widely used, while benchmark datasets have also been proposed. Specifically, datasets used by benchmark studies reviewed herein have largely been real, experimental datasets investigating a particular phenotype (i.e. the object of study in the experiment). Following Tarca *et al.* [23], several studies [2, 3, 9, 13, 25] have selected evaluation datasets as those which correspond to a pathway or gene set from the chosen database (e.g. dataset investigating the breast cancer versus normal phenotype and the breast cancer pathway). Others [14, 16, 24] have focused on measuring consistency across methods by selecting various datasets that study the same phenotype. Finally, comparative studies [3, 13, 14, 18, 22] have also employed simulated datasets to benchmark methods as various features of the data can be tuned and the method can be studied under these known features of the data. In line with Tarca *et al.* [23], the majority of studies have evaluated the performance of an enrichment method on these datasets based on at least one of the following metrics: prioritization, specificity or sensitivity.

Prioritization is evaluated based on whether a target gene set that has been identified a priori as showing high relevance to a phenotype associated with the dataset under investigation is ranked near the top (e.g. the breast cancer pathway is expected to hold the topmost ranking for a dataset measuring transcriptomic differences between the breast cancer versus normal phenotypes). Specificity refers to the proportion of gene sets that are correctly identified by a method as true negatives; thus, methods with a high specificity will generate fewer false positives. Finally, of all the gene sets detected as significant by a given method, sensitivity measures the proportion of gene sets that are actually relevant to the phenotype associated with the dataset under study (i.e. true positives).

Of the various comparative studies done to date, the above-mentioned metrics have been among the most commonly used for the empirical evaluation of enrichment methods. Nonetheless, the metrics used and the methods benchmarked by an individual study can vary greatly, with the most popular methods, not surprisingly, studied the most frequently. Yet despite the numerous benchmark studies conducted thus far, a comprehensive and standardized assessment of the many enrichment methods available has yet to be performed. Moreover, of the benchmark studies that have attempted such an assessment, no specific method has been shown to yield consistent results across all evaluated settings. Nevertheless, trends do emerge regarding the individual performance of a method on a given metric (Supplementary Tables 4–6 available online at https://academic.oup.com/bib). Thus, in the following, we report the trends observed across comparative studies for methods that consistently show superior performance on metrics in two or more studies without showing a poor performance on that same metric.

With regard to sensitivity, MRGSE [26], GlobalTest [27] and PLAGE [28] ranked highly in studies by Tarca *et al.* [23] and Zyla *et al*. [25] (Supplementary Table 4 available online at https://academic.oup.com/bib). However, high sensitivity may also imply a lower specificity. This was indeed observed for MRGSE and PLAGE, both of which reported a larger than expected number of false positives in at least one study, though also a good performance in prioritization (Supplementary Table 6 available online at https://academic.oup.com/bib). This is not surprising given that both methods have also been shown to report a majority of gene sets as significant [24, 25]. Similarly, classical statistical tests, including the KS test and the Wilcoxon rank sum test, were highly sensitive in Bayerlová *et al.* [13] and Nguyen *et al.* [9], though results were inconsistent regarding their specificity. Notably, of the above-mentioned methods, GlobalTest was the only investigated method to consistently demonstrate high sensitivity as well as high specificity in studies by Tarca *et al.* [23] and Zyla *et al*. [25].

In assessments of specificity, SPIA [29] and CAMERA [30] have shown high specificity in at least two studies (Supplementary Table 5 available online at https://academic.oup.com/bib), though results have been mixed or poor with regard to sensitivity and target pathway prioritization. Furthermore, GSA [31], PADOG [32] and PathNet showed good results with regard to prioritization (Supplementary Table 6 available online at https://academic.oup.com/bib) but mixed results for sensitivity and specificity. Finally, across all studies, GSEA [33] and ORA (or a variant) were the most investigated enrichment methods, with 8 of 12 comparative studies assessing either one or both of these methods (Supplementary Table 3 available online at https://academic.oup.com/bib). Here, we observed that, although they were the most commonly used methods for enrichment analysis, results regarding their sensitivity, specificity and prioritization were altogether inconsistent (Supplementary Tables 4–6 available online at https://academic.oup.com/bib).

### Hypothesis testing and significance assessment

Much of the focus of comparative analyses on gene set analysis methods has been on the implications of alternative definitions of the null hypothesis. In their seminal work, Goeman and Bühlmann [34] characterized methods by the null hypothesis assumed in the statistical test. Enrichment methods, they assert, can be categorized as being competitive methods if they test the competitive null hypothesis [i.e. those which assume that genes in a gene set are not differentially expressed with respect to their complement (typically the rest of the genes in the experiment)] or self-contained methods if they test the self-contained null hypothesis (i.e. those which assume that genes in a gene set are not differentially expressed across phenotypes). Choosing one category of methods over another can confer several advantages, which we explicate through a brief review of studies that have assessed the performance of methods, which differ based on this distinction.

Rahmatallah *et al.* [22] recapitulated earlier work [35–37], generally noting that the power of self-contained methods was greater than that of competitive ones (Table 1; Supplementary Tables 2 and 3 available online at https://academic.oup.com/bib). Self-contained methods were also more robust to sample size and heterogeneity, with these methods showing the highest sensitivity among all the ones they evaluated, even as the sample sizes decreased [22] (Supplementary Table 7 available online at https://academic.oup.com/bib). Specifically, they found that ROAST [38] and SAM-GS [39] yielded the best performance on this metric.

Geistlinger *et al.* [2] noted that the proportions of gene sets reported as significant by methods differed based on the type of null hypothesis tested. Out of 10 investigated methods (Supplementary Table 3 available online at https://academic.oup.com/bib), they found that the majority of self-contained ones, including GlobalTest, detected a larger fraction of gene sets as significant. In Zyla *et al.* [25], the self-contained methods GlobalTest and PLAGE also reported the largest number of gene sets as significant among all benchmarked methods (Supplementary Table 3 available online at https://academic.oup.com/bib). In contrast to these findings, Wu and Lin [37] found that GlobalTest reported fewer gene sets as significantly enriched in comparison with competitive methods.

Furthermore, Geistlinger *et al.* [2] found that self-contained methods, particularly GlobalTest and SAM-GS, were especially sensitive to gene set size, with a propensity toward detecting larger gene sets as significant (Supplementary Table 8 available online at https://academic.oup.com/bib). For example, even when random gene sets were assembled, GlobalTest and SAM-GS identified all gene sets with over 50 genes as significant. However, Maleki *et al*. [16] noted that GlobalTest was among the methods more likely to identify gene sets of smaller sizes as significant (Table 1; Supplementary Table 3 available online at https://academic.oup.com/bib), albeit, in this case, the upper bound for genes in a given gene set was nearly 2000, while in Geistlinger *et al.* [2], it was 500.

These contradictory findings are a prime example of the challenges associated with benchmarking methods for gene set analysis. Such glaring variability in results yielded by the same method investigated in different studies may be due to several factors, such as gene set size or differing proportions of DEGs in the studied datasets. For instance, GlobalTest tends to perform sub-optimally when only a few genes in a given gene set are differentially expressed and the majority of genes are not, and it conversely tends to be better suited for when there are many genes with small changes in differential expression in a gene set [37, 40]. We further discuss the impact of gene set size on results in a subsequent section as well as in Supplementary Text 1 (available online at https://academic.oup.com/bib).

If opting to select a competitive method instead, one must consider that testing the competitive null hypothesis often inherently implies the intended association not only between the phenotype and the genes within a given gene set but also between the phenotype and the genes in the complement of the set [40]. That said, competitive methods can be appropriate when the goal is to test for excessive amounts of differential expression among genes in a gene set. For instance, the popular ORA method was noted as suitable when there are large levels of differential expression [2]. However, ORA also tends to prioritize larger gene sets, assigning them lower *P-*values [16, 23]. Nonetheless, in Geistlinger *et al.* [2], ORA and other competitive methods outperformed the self-contained ones in ranking phenotype relevant gene sets near the top (Supplementary Table 9 available online at https://academic.oup.com/bib). In contrast, although ORA performed favorably on the prioritization of relevant gene sets in Tarca *et al.* [23], no clear discernment could be made with regard to the performance of competitive and self-contained methods on this measure (Supplementary Table 6 available online at https://academic.oup.com/bib). Furthermore, while self-contained methods tended to identify a larger proportion of gene sets as significant in Geistlinger *et al.* [2], the majority of competitive methods (i.e. SAFE [41], GSEA, GSA and PADOG) did not identify any significant gene sets.

Intimately linked to the formulation of the null hypothesis is the calculation of the *P*-value [34]. Divergent approaches to assign a *P-*value to a gene set address the following question: What is the sampling unit? If the sampling unit is the gene, for each gene set of a given size, an equal number of genes are randomly drawn from all genes under investigation to sample the null distribution. If, however, the sampling unit is the subject, the phenotypic labels of subjects are randomly permuted to sample the null distribution instead. While methods that test a self-contained null hypothesis are generally linked with sample permutation and competitive methods with gene permutation, the latter group of methods can be modified to make them self-contained [40].

Sample permutation is often regarded as the preferred approach to obtain the empirical null distribution as its setup tends to pertain more naturally to the research question at hand of whether or not an association exists between a gene set and a phenotype. In contrast, methods that calculate significance by gene permutations suffer from the assumption that genes are independent and identically distributed (iid). It is well established, however, that this premise does not hold true in a real biological context where gene correlations (i.e. the coordinated expression of genes) can be observed and where sets of genes are known to work in tandem [37]. Thus, in the case of gene permutations, while significant gene sets may be reflective of either gene correlations that arise regardless of experimental condition and/or actual phenotypic differences, it is the latter that is often far more interesting, and the former can inflate the number of false positives [37, 40, 42, 43].

The effects of correlations within gene sets have been observed in various studies. Tamayo and colleagues [44] show that these correlations can have major implications on the results of enrichment analysis by comparing the results of GSEA against a simple parametric approach in 50 datasets. They observed that the parametric approach, which assumes differential gene expression scores are both independent and follow a normal distribution, yields a larger number of significant gene sets than GSEA, but many of these are speculated to be false positives. Similarly, in experiments on simulated data in Maciejewski [40], the author demonstrated that when gene correlations were present in the gene set yet there were no DEGs either in the gene set or its complement, false positive rates for methods that make the iid assumption (e.g. parametric methods proposed in Irizarry *et al.* [45] and competitive methods with gene permutation) were greater than expected. Thus, the authors of these studies caution that methods that assume gene independence may report gene sets as significantly associated with a phenotype when in fact gene correlations account for the purported, significant results. However, it is also worth noting that the influence of correlations can be somewhat mitigated by reducing redundancies within gene sets.

In Maciejewski [40], the author observed that among methods with a sample permutation procedure, GlobalTest, GSEA and GSA and its variant achieved higher power. Furthermore, GSEA, a competitive method with sample permutation, had higher power than several other methods tested (i.e. GSA and its variant, PAGE [46], Wilcoxon rank sum test, Q1 [47] and SAFE), although as the number of DEGs in a gene set increased, so too did the power of the other methods.

Nevertheless, sample permutation requires an adequate number of samples as without a sufficiently large sample size, the calculated *P*-value may never achieve significance, in which case, gene permutation is recommended. For instance, in their comparative analysis, Maleki *et al.* [48] found that, across 10 replicate datasets, GSEA with sample permutation was unable to detect any gene set as enriched when sample sizes were small, suggesting a lower bound of 10 samples for this particular method. The robustness of various methods to changes in sample size is further discussed in Supplementary Text 2 (available online at https://academic.oup.com/bib).

Other methods have been proposed that attempt to address some of the drawbacks associated with sample and gene permutation approaches by conducting both sample permutations and gene randomizations in a method known as restandardization, as with GSA, through the use of rotations for gene set testing, as with FRY [49] and ROAST, or via bootstrapping methods, as in Zahn *et al.* [50] and Barry *et al.* [43].

### Topology- and non-topology-based methods

Methods for enrichment analysis can also be classified as those which are topology-based or non-topology-based. The latter group of methods can be further sub-classified into the aforementioned ORA and FCS methods, the so-called first- and second-generation approaches, respectively [1]. PT- or topology-based methods fall into the category of third-generation approaches, intuitively more advanced as, unlike ORA and FCS methods, they leverage the topological structure of genes in a pathway. Nonetheless, results from multiple benchmarks on topology- and non-topology-based methods are inconclusive as to the superiority of one group of methods over another, with studies suggesting topology-based methods have the upper hand.

In Bayerlová *et al.* [13], authors noted that whether a method was topology-based or not was inconsequential to performance when original KEGG pathways (which tend to contain overlapping genes) were used in experiments (Supplementary Tables 3–6 available online at https://academic.oup.com/bib). Notably, while CePa includes pathways from both KEGG and the Pathway Interaction Database [51], other topology-based methods evaluated in the study (i.e. PathNet and SPIA) are only compatible with pathways formatted in a custom-XML format (i.e. KEGG Markup Language). This result is particularly striking, considering KEGG contains overlapping pathways, thus limiting the potential of topology-based methods by restricting users to pathways formatted in the manner specified by this database. In contrast, experiments done using non-overlapping pathways resulted in topology-based methods outperforming non-topology-based ones [13]. In line with these findings, comparative studies by Jaakkola and Elo [14] and Nguyen *et al.* [9] similarly suggested that topology-based methods exhibit an improved performance over non-topology-based ones under certain conditions, albeit, contrary to findings by Bayerlová *et al.* [13], these conclusions were drawn exclusively using KEGG as the choice of pathway database.

More particularly, results from Nguyen *et al.* [9] indicate that topology-based methods have a slight upper hand in detecting target pathways as compared to non-topology-based ones (Supplementary Table 6 available online at https://academic.oup.com/bib), though results were mixed regarding the *P*-values of target pathways. In Jaakkola and Elo [14], topology-based methods (i.e. SPIA, CePa and NetGSA [52]) detected a larger number of significant pathways than non-topology-based ones (i.e. GSEA, Pathifier [53] and DAVID [54]). However, in a more challenging dataset where differences across groups were subtle, nearly all studied methods identified either no pathways or relatively few pathways as significantly enriched.

Ihnatova *et al.* [3] conducted several experiments, which assessed the influence of various parameters on topology-based methods [e.g. sensitivity to pathway and sample size (Supplementary Table 7 available online at https://academic.oup.com/bib), specificity (Supplementary Table 5 available online at https://academic.oup.com/bib) and exclusion of topological information]. As a proxy to study the latter parameter (i.e. whether topological information affects results for a given topological method), the authors evaluated the influence of single genes on the fraction of pathways that were considered enriched, assuming that a setup that fails to take into account PT is one in which individual genes have an equal impact on results. To that end, they found that TopologyGSA [55] and Clipper [56] yielded no difference in performance when topological information was excluded, while for all other methods, the exclusion of topological information led to the identification of a smaller fraction of enriched pathways. In addition, in assessing whether the ranks/*P-*values of target pathways change when topological information is incorporated, the authors found that both the ranks and *P-*values of target pathways decreased for PRS [57] and CePa, while for all other methods, the inclusion of topological information resulted in either no change or an increase in ranks/*P-*values of target pathways (at times caused by pathway-specific effects).

### Additional methodological considerations and consensus approaches

Besides the above-mentioned common measures and classifications, several comparative studies have used to draw distinctions between enrichment methods, the performance of methods on a number of additional aspects has also been benchmarked. We refer to the studies that evaluate other aspects, including accuracy (Supplementary Table 10 available online at https://academic.oup.com/bib), type I error rate, power, runtime and assessments of reproducibility across datasets, among others in Supplementary Table 11 (available online at https://academic.oup.com/bib). Furthermore, we outline additional methodological considerations, including the steps used in data preprocessing and biases, which arise from experiments (Supplementary Text 3 available online at https://academic.oup.com/bib), the gene- and gene set–level statistics selected (Supplementary Texts 4 and 5 available online at https://academic.oup.com/bib), the applicability of enrichment analysis to various omics dataset types (Supplementary Text 6 available online at https://academic.oup.com/bib) and the choice of background (Supplementary Text 7 available online at https://academic.oup.com/bib).

Given the vast variety of enrichment methods, often with tunable settings, hundreds of methods and variants are at the disposal of life science researchers. As results can acutely vary according to the method selected, such a broad variability has prompted the development of tools to conduct enrichment analysis in concert. While the techniques to do so can differ, generally a consensus is taken across several methods to determine the final set of pathways that are interesting in some statistically significant way. Examples to do so include the R packages EGSEA [58], EnrichmentBrowser [59], Piano [60] and decoupleR [61] as well as the ML-based approach, CGPS [62] and the CPA web application [63]. Details on each of these ensemble techniques are provided in Supplementary Text 8 (available online at https://academic.oup.com/bib).

## Impact of pathway database and gene set size

While variations of enrichment methods have been among the most studied factors that influence the results of an enrichment analysis, there are several other considerations to be made in the design of an experiment to ensure biologically meaningful results. In this section, we introduce studies, including notable benchmarks, that have investigated the impact of additional factors on the results of enrichment analysis, such as database choice and pathway size.

One of the most critical factors the results of an enrichment analysis can hinge upon is the choice of a reference pathway database(s). It is common practice for researchers to solely rely upon a single database for an enrichment analysis, which can be due, in part, to a researcher’s preferences, the popularity of a particular database or its ease of usage, among other factors. Indeed, we observed that the majority of studies that benchmarked the performance of enrichment methods (Table 1) were almost always conducted on a single database, and that too, primarily KEGG.

A first investigation on the importance of selecting a collection of gene sets was performed by Bateman *et al.* [64]. In this study, the authors demonstrated how the seven standard collections housed within MSigDB yielded different results when conducting GSEA within the context of a drug response cancer dataset. Among other findings, the results of this study indicated that some collections were able to yield a significantly larger number of enriched pathways relevant to the studied phenotype than others. Furthermore, the authors argued that the choice of gene set collections should not be made arbitrarily as certain gene sets may be more or less suitable for a particular dataset than others. In a recent study on best practices for the popular ORA method on metabolomics data [65], the authors also found that the results of pathway analysis substantially differed based on the choice of pathway database (i.e. KEGG, Reactome and BioCyc [66]).

Similar conclusions were drawn in our previous work [67] in which we evaluated whether enrichment results are in consensus for any given pathway that can be found across three major pathway databases (i.e. KEGG, Reactome and WikiPathways [68]) and multiple enrichment methods. Our study revealed the advantages of combining multiple databases by using equivalent pathway mappings, demonstrating that an integrative resource can yield more consistent results than an individual one. Overall, these studies demonstrate the importance of database choice, a crucial factor given the differences in coverage across databases [69, 70]. Finally, we would also like to note the importance of database size as the total number of pathways present in a database has an influence when multiple correction methods are applied.

An additional factor that is related to database choice is gene set (pathway) size, corresponding to the number of genes within a gene set for enrichment methods that do not consider PT, or the number of nodes (genes) and edges for those that do consider it. The effect of pathway size has recently been studied in Karp *et al*. [71] by comparing the significance of six equivalent pathway definitions from KEGG and EcoCyc [72]. Given the differences in the average size of a pathway across the two databases (i.e. KEGG pathways are significantly larger than their respective homologs in EcoCyc), the authors investigated the degree to which size could influence results, finding that pathway size can have a stronger effect than the statistical corrections used. Furthermore, the authors found that KEGG pathways required up to two times as many significant genes in order to attain the same *P*-value as their EcoCyc counterparts.

Notably, size differences between equivalent pathways have not only been examined for these two databases but also across other major resources, such as Reactome, and WikiPathways. In this work, the authors argue that using pathway definitions that span across several biological processes (e.g. signal transduction) can lead to misinterpretations as when these pathways are enriched, it is difficult to construe whether this implicates all or only a subset of the pathway. These broadly defined pathways can also be less informative, contributing little in terms of novelty to the overall understanding of the distinctions between the phenotypes under study. Nonetheless, smaller pathways can lead to exceedingly long results and overly strict multiple testing corrections [4].

Possible solutions for mitigating the impact of gene set size on results are defining the minimum and maximum number of genes within a gene set (e.g. between 10 and 500), careful consideration of the enrichment analysis method selected (see ‘Hypothesis testing and significance assessment’ section) as well as addressing redundancies within gene sets, as proposed in [73]. In their approach, the authors suggest discarding significant gene sets that overlap with others in order to ensure that the enrichment of a particular pathway is not a result of the overlay.

While database choice and pathway size are two critical factors to consider, we foresee several approaches to offset the challenges they create. In the case of database choice, a study by Maleki *et al*. [74] proposed two simple metrics (i.e. permeability and maximum achievable coverage scores) to assess the degree of overlap between a gene list of relevance and all gene sets within a database. The goal of these metrics is to provide an intuition of whether or not the genes of a phenotype under investigation are well covered by a particular database. Thus, the authors argue that this approach can reduce database bias and arbitrary database selection as the two scores can guide users to rationally decide upon the most appropriate database.

Another solution that we propose is that the enrichment results generated from a reference database could be validated against an additional database using equivalent pathway mappings across them. By leveraging pathway mappings, one can assess the similarity between the results obtained from different databases (i.e. reference and ‘validation’ database) to confirm whether they are in consensus, or re-evaluate them if they are not. In earlier work, we leveraged this technique by generating equivalent pathway mappings across four pathway databases [75]. A web tool (i.e. DecoPath) subsequently enables users to evaluate similarities and differences at the gene and pathway level for a given pathway across databases and enrichment methods. For instance, a particular pathway in one database can have a slightly different gene set than the same pathway in another database, which can ultimately explain why a pathway is detected as significantly enriched in one database but not in another.

Similarly, pathway mappings can also be employed to systematically study the impact of pathway size on results. Here, one could leverage hierarchical mappings (i.e. pathway A is part of pathway B) from pathway ontologies to evaluate whether related pathways are similarly enriched. Although a pathway ontology was earlier proposed by [76], it has neither been adopted by nor linked to any major database. Instead, each database utilizes its own pathway terminology, though some databases such as Reactome and GO also incorporate a hierarchical organization within their schema. In fact, Reactome recently adopted such an approach to facilitate the interpretation of enrichment analyses through implementing ReacFoam, a visualization for navigating through its pathway hierarchy and exploring the degree of enrichment of pathways at different levels.

The growth of biomedical literature is reflected in pathway databases as their pathway definitions change over time. A study by Wadi *et al*. [77] demonstrated the impact of outdated pathway definitions in several web-based tools as well as highlighted that the number of pathways/biological processes doubled in 7 years (2009–16) in major resources such as Reactome and GO. Furthermore, it revealed that the majority of the studies analyzed were conducted using outdated pathway definitions, constituting a major issue as the results presented in such studies could have potentially changed. We believe this problem can be partially mitigated if users are alerted by pathway enrichment tools when the underlying pathway database(s) has not been recently updated. Furthermore, updating the information from pathway databases in a tool has been greatly simplified by the APIs and services offered by major resources such as Reactome, GO and WikiPathways. Finally, we encourage researchers to include both the version of the database(s) used in the analysis as well as the version of the tool(s) employed.

## Impact of additional factors on enrichment analysis and possible future benchmarks

While the factors mentioned thus far have each been benchmarked with regard to their impact on pathway enrichment results, there exist other factors that have not yet been explored in detail. First, at a more granular level, individual genes can also have an impact on results. A study by Ballouz *et al.* [78] raised the challenges associated with annotation bias and redundancies in gene sets. The annotation of a single gene to many functions (i.e. multifunctional genes) can potentially confound the results of a pathway analysis as these genes may result in a sizeable number of enriched pathways that are largely irrelevant. For example, several pathways with multifunctional genes may be considered enriched in the results, though the enrichment of these pathways could be due to the presence of multifunctional genes rather than the relevance of the pathway to the phenotype of interest. One approach the authors propose to control this effect is by performing repeated runs of the analysis while removing the topmost multifunctional genes in the dataset in order to identify the most robust pathways. Furthermore, other ways to reduce the effect of multifunctional genes can include assigning weights to genes based on their promiscuity, though this approach might also have drawbacks.

A second factor that has not yet been investigated, which is related both to database updates and choice, is the size of a database measured by the number of pathways. This factor is not only important due to its correlation with the coverage of biological processes but also because the size of the database can influence the significance of the results when correcting for multiple testing (see Supplementary Text 9 available online at https://academic.oup.com/bib). As a consequence, depending on the size of a database, the same pathway in one database may or may not be enriched in another after applying multiple testing correction. This is often the case when comparing popular databases, such as KEGG and Reactome, whose number of pathways can differ by an order of magnitude.

Finally, we would like to note that there are other interesting factors, which could potentially be analyzed in the future. First, for topology-based methods, the particular network structure of some pathways may make them more susceptible to enrichment than others given the topological differences identified by [79]. Thus, one future possible benchmark could investigate the effect of network sparsity on pathway enrichment, or if hubs within a network correlate with greater enrichment. Second, another factor to evaluate is the degree to which a bias toward certain indications in pathway knowledge influences results. For example, there is an over-representation of interactions characterized in widely studied indication areas, such as cancer [80, 81], and thus, pathways containing these interactions may appear in the results of enrichment, while possessing little relevance to the studied phenotype. To investigate this factor, resources such as BioGrid [82] where protein–protein interactions are annotated with experimental metadata can be leveraged since the majority of databases do not provide information on the provenance supporting each interaction. Third, only a minute fraction of known proteins have been experimentally annotated with functional characterizations, while functional annotations for the vast majority of proteins are either inferred, presumptive or unknown [83, 84]. Several computational methods exist for protein function prediction, and while such methods are routinely benchmarked [85], the effect of experimental versus predicted functional annotations of proteins on downstream analyses also warrants further study. This is of particular importance to GO enrichment, where numerous algorithms have been developed to predict GO terms for proteins [86].

## Discussion

The last decade has seen an explosion in the usage of pathway enrichment analysis, spearheaded by both an abundance in the volume of available data and the interpretive power of these analyses [10]. Prompted by a wide range of available enrichment methods and pathway resources, several comparative studies have evaluated how different factors can influence the results of such an analysis. Here, we have reviewed the findings of these studies in order to provide a comprehensive overview on the impact of these factors. Furthermore, we have suggested possible approaches to overcome some of the limitations discussed as well as possibilities for additional benchmark studies on other, under studied factors.

In the first section of this review, we have outlined the results of 12 comparative studies that have investigated differences across pathway enrichment methods. Many of these studies have specifically focused on the performance of individual methods on popular metrics (e.g. prioritization, sensitivity and specificity), keeping in mind that without gold standards to conclude whether the results from any given method are biologically sound, objective evaluations can be difficult to make. Overall, we have found many inconsistencies in the performance of methods across metrics as well as across studies. While there is no consensus across studies on whether a specific method outperforms others, we have reported trends we have observed regarding the top-performing methods for each metric.

Though we note that the performance of the majority of methods on these and other metrics is inconclusive, whether a particular method is a reasonable choice for a certain use case can depend on a number of factors, such as the goal of the experiment, the dataset in question or particulars of the gene set collection. Nevertheless, trade-offs between performances on certain metrics can be important considerations in the selection of a method. For example, given a dataset where changes in differential gene expression between experimental groups are subtle, a highly sensitive method can increase the likelihood of detecting a signal. Thus, a large number of gene sets that are truly significant can be identified, essentially ruling out nearly all gene sets that are not detected, albeit at the expense of producing a greater number of false positives. If, however, changes in differential gene expression between experimental groups are generally more pronounced, a method ranked high in specificity may be preferable to preclude the detection of too many gene sets, which can complicate interpretation.

We have also examined comparative studies that have evaluated the differences between distinct categories of enrichment methods, such as how the null hypothesis is formulated and the sampling unit is defined, noting that the selection of one category of methods over another can have serious repercussions on the fraction of gene sets that are significant and their ranks. In addition, a major categorical distinction is drawn between topology- and non-topology-based methods, which have been reviewed in several benchmarks. We have found that, though topology-based approaches are more advanced, for some methods, the removal of topological information yields no differences in results, for other methods, it can improve results, and several are constrained in that they only cater to KEGG pathways (or pathways in an equivalent format). Finally, we reviewed studies that have assessed the influence of particular, modular aspects of a typical enrichment analysis as well as outlined additional aspects one must be cognizant of that can affect the behavior of a given method, which ultimately reflects in the overall results of an analysis.

We have reviewed several other factors apart from enrichment methods, such as pathway size and database choice. Notably, the latter can be subjective, with both researcher preferences and distinct research goals taking precedence over set guidelines. However, we have outlined approaches that leverage pathway mappings to mitigate the effect of these factors. An additional aspect discussed in this review is the lack of regular updates to enrichment tools, which reflect updates made to pathway databases. Fortunately, this issue has, at least, partially been addressed by the adoption of API services by major pathway resources. Nevertheless, the amount of literature published on a daily basis continues to grow, making the task of maintaining up-to-date pathway definitions difficult, particularly for public and academic resources. Thus, we envisage that the path forward to address this shortfall is to improve interoperability across databases via mappings [70] or through the use of common database formats [87].

Finally, we would like to mention possible future benchmarks beyond the ones we have previously proposed. First, future benchmarks can benefit from the existence of a gold standard prioritization approach, for instance, one that leverages well-established pathway-disease associations from genetic disorders, similar to the assessment proposed in [9], which exploits knockout datasets. Second, given the rise of multi-omics datasets, we anticipate the development of enrichment methods that operate on other modalities beyond mRNA data, such as metabolomics (see Supplementary Text 6 available online at https://academic.oup.com/bib). Last, we foresee that the insights gained from multi-omics experiments will also be reflected in pathway definitions in two ways: (i) the appearance of ‘dynamic pathways’ (i.e. contextualized pathways representing particular pathway states as opposed to general, static diagrams) and (ii) a shift from traditional gene sets to sets of multimodal biological entities.

## Conclusion

In conclusion, the effect of various factors on pathway enrichment analysis is apparent. Numerous studies have demonstrated how variations in the design of an enrichment analysis can lead to altogether different findings. At the extremes, comparative studies have shown how certain experimental setups can detect either all or no gene sets as interesting in some statistically significant way. We summarize the key findings of studies reviewed herein as follows:

### Formulation of null hypothesis and significance assessment

One must be cognizant of how the null hypothesis is formulated (i.e. competitive or self-contained) as methods categorized into one or another approach behave differently in terms of the fraction of gene sets reported as significant, as well as their sensitivity to gene set size, sample size and sample heterogeneity. Self-contained methods also tend to have greater power than competitive methods and careful consideration should be made taking into account the proportion of genes that are differentially expressed in the dataset. Similarly, in order to calculate a *P-*value for each gene set, one must bear in mind that disparate approaches can impact the results of an enrichment analysis, and depending on the approach taken, introduce false positives.

### Pathway and sample size considerations

Certain enrichment methods have been observed to be more or less robust to pathway and sample size than certain others. Sensitive methods may detect larger gene sets as significantly enriched and their sensitivity can be tied with whether they are competitive or self-contained methods. Not surprisingly, a method’s performance tends to deteriorate with decreasing sample size, although some methods are more robust on this factor than others.

### Topology- versus non-topology-based methods

Topology-based methods are intuitively more advanced than non-topology-based ones. Incorporation of topological information tends to improve the ranks and *P-*values of relevant pathways for some topology-based methods, yet this may not be the case for all. Nonetheless, some topology-based methods are limited or at least partial to specific pathway databases.

### Choice of gene set collection or pathway database

The selection of one gene set collection over another can lead to different results. Some collections or databases may be more suitable than others for a given dataset. The selection of a database is complicated by variable definitions of pathway boundaries as well as by redundancies and outdated pathway definitions.

The errors from these steps that propagate through an enrichment analysis may be inconsequential at best and misleading at worst. Although there is no singular method or gene set collection/pathway database, which is advisable for enrichment analysis over all others, well-informed choices can be made and solutions to mitigate the impact of various factors are available. Furthermore, recently, many ensemble approaches have been developed so that users can benefit from multiple databases and/or methods.

**Key Points**

- Pathway enrichment analysis is a widely used technique for the interpretation of biological data
- In recent years, the advent of a multitude of enrichment methods and pathway databases has led to several benchmarks to study the impact of various factors on the results of enrichment analysis
- This review outlines key aspects of enrichment analysis and summarizes results of studies, which have evaluated their influence
- We propose solutions to mitigate the effect of these factors and identify possible future benchmarks

## Authors’ contributions

S.M. and D.D.-F. wrote the manuscript. A.T.K. and M.H.-A. reviewed the manuscript. All authors have read and approved the final manuscript.

## Supplementary Material

Review_Supplement_bbac143

Methods_Benchmark_Supplement_bbac143

## Author Biographies

**Sarah Mubeen** is a doctoral student at the University of Bonn and is a research fellow at the Department of Bioinformatics at the Fraunhofer Institute for Scientific Computing and Algorithms (SCAI). Her research interests include pathway and network-based approaches for the interpretation of biomedical data.

**Alpha Tom Kodamullil** is the group lead of Applied Semantics at the Fraunhofer Institute SCAI. Her main research focuses on knowledge graphs around diseases and shared semantics, which lay the foundation of data and knowledge interoperability.

**Martin Hofmann-Apitius** is a professor at the Bonn-Aachen International Center for Information Technology at the University of Bonn and is the head of the Department of Bioinformatics at the Fraunhofer Institute SCAI. His research interests involve integrative semantics, natural language processing, data- and knowledge-integration in neurodegeneration research and longitudinal modeling of disease progression.

**Daniel Domingo-Fernández** is a research fellow at the Department of Bioinformatics at the Fraunhofer Institute SCAI and is a senior bioinformatician at Enveda Biosciences. His research interests lie in integrating a priori knowledge and biomedical data for drug discovery, predictive modeling and patient stratification.
