# Supplementary material for: On the influence of several factors on pathway enrichment analysis
Source: Brief Bioinform. 2022 Apr 23;23(3):bbac143. doi: 10.1093/bib/bbac143 (PMC9116215; doi:10.1093/bib/bbac143)
Supplement: Review_Supplement_bbac143 [file review_supplement_bbac143.docx]

# **Supplementary File**

1. **Sensitivity to gene set size**

Many of the studies we review **(see Table 1)** have also compared the performance of enrichment methods by their sensitivity to gene set size **(Supplementary Table 8)**. Across studies, little consensus could be noted on this factor, complicated by variability in how set sizes are binned and definitions of what constitutes, for example, a large gene set. For instance, in Ihnatova *et al.* [1]*,* large pathways in their studies were those that contained >= 35 genes (and up to 344), in Bayerlová *et al.* [2], that number was over 80 (and up to 380), whereas the sizes of gene sets in Geistlinger *et al.* [3] and Maleki *et al.* [4] reached 500 and nearly 2,000, respectively. Thus, objective comparisons can be difficult to make, and here instead, we briefly discuss study-specific trends related to this parameter.

In Maleki *et al.* [4], the authors observed that some methods tended to preferentially report gene sets of larger sizes as significant (i.e., FRY [5], ROAST [6], GlobalTest [7] and GSEA-G [8]), while others tended to report smaller ones (i.e., GAGE [9] and ORA). Though it may be the case that a method shows poor robustness against this parameter, a method itself may still be well-suited for gene sets within a specific range of sizes. For example, although ORA performed suboptimally for smaller gene sets in Tarca *et al.* [10], both ORA and GSEA-G ranked as the top methods when relevant gene sets were larger in size. Additionally, studies by Ihnatova *et al.* [1] and Ma *et al.* [11] compared topology-based methods on this metric. Overall, larger pathways were found to have smaller *p*-values for all topology-based methods investigated, except PRS [12] in Ihnatova *et al.* [1], while Ma *et al.* [11] assessed their performance with respect to gene expression as well as metabolomics/lipidomics data, a comparison closely related to pathway size (i.e., focus is on smaller pathways in the latter case). The authors found that all investigated methods generally tended to perform about the same in the case of large pathways and their previously published method, NetGSA [13], as well as DEGraph [14], held an upper hand over other methods in the case of small biochemical pathways. Due to the substantial implications of gene set size on the results of enrichment analysis, this factor is further discussed in a subsequent section.

1. **Sensitivity to sample size**

Several studies have specifically investigated the impact of sample size on method performance. Not surprisingly, performance tends to decay as sample sizes decrease, though some methods tend to be more robust than others with regard to *p-*values of target pathways [15], the true positive rate [16], and proportions of enriched pathways [1]. A survey of the performance of methods based on their robustness to sample size can be found in **Supplementary Table** **7**. Here, again, we describe the trends noted across studies concerning the performance of methods (i.e., those which show good performance in at least two studies), finding MRGSE [17], ORA, GSEA with gene permutations (GSEA-G) and PADOG [18] to be among the non-topology based methods with consistently good performance. The influence of sample size was also investigated specifically for topology-based methods in Ihnatova *et al.* [1] with regard to the proportion of enriched pathways detected. Here, SPIA [19], PRS, and CePa [20-21] were the most robust against this factor. By contrast, TopologyGSA [22], Clipper [23], DEGraph, and TAPPA [24] were highly sensitive to increasing sample size [1].

In Maleki *et al.* [25], the authors note that the reproducibility of a method suffers when groups contain fewer than five samples. Furthermore, for some methods (i.e., GSVA [26], GAGE, FRY, and ROAST), they observed that an increase in sample size tends to be accompanied by an increase in the number of significant pathways, though this likely implies a greater number of false positives. In contrast, PLAGE [27] and ssGSEA [28] detected nearly all gene sets as enriched regardless of the sample size. As these latter two methods were found in studies by Tarca *et al.* [10] and Zyla *et al.* [15] to be highly sensitive **(Supplementary Table 4)**, these results may not be altogether surprising.

1. **Data preprocessing**

Another important consideration in performing enrichment analysis is in the selection of the steps used in data preprocessing. For microarray data, these steps include background correction to remove noise, and normalization to reduce variations from biological and technical factors that can occur in the measured intensities of gene expression [29]. Methods used for major preprocessing steps include RMA [30], its variant, gcRMA [31], MAS 5.0 (Affymetrix; version 5), and variance stabilizing normalization (VSN) [32-33]. With regard to the preprocessing of RNA-seq data, several different approaches have been benchmarked in a comprehensive review by [34]. Here, the authors contend that the normalization method selected can substantially impact downstream analyses, finding that DESeq [35] and TMM [36] generally display good performance across a variety of measures whilst relatively poor performances were noted for RPKM [37] and Total Count [34]. Notably, however, normalization may not be able to address certain biases which arise in RNA-seq experiments. For instance, long and highly expressed transcripts tend to be over-represented in RNA-seq data and can bias the results of downstream analyses [38].

1. **Gene – level statistics**

Depending on whether an enrichment method falls into the category of a univariate or multivariate approach, a variety of gene and/or gene set –level statistics can be selected. While multivariate approaches skip this step, in univariate approaches, a gene-level (i.e., local) statistic is first used to measure differences in gene expression levels and calculate scores for each gene in the dataset [39]. In Ackermann and Strimmer [40], the authors proposed a modular framework for enrichment analysis which they then used to compare 261 different variants on 10 simulated and 2 experimental datasets. In this study, the authors compared the following gene-level statistics: two-sample t-statistic, moderated t-statistic and Pearson correlation coefficient. Here, they concluded that the gene-level statistic used has little contribution to the overall rankings of the gene sets. By contrast, they noted that transformations of the gene-level statistic (i.e., squared values, ranks, local false discovery rate, binary transformations or none) do have an impact on the overall results, including the number of gene sets detected as significantly enriched. For instance, a transformation (e.g., absolute or squared value of the gene score) may be essential to detect gene sets that contain genes altered in opposite directions by a phenotype (i.e., both upregulated and downregulated).

Later investigations on the choice of gene-level statistic were conducted by Zyla *et al.* [41] where they evaluated the performance of 16 different ranking metrics in GSEA, a popular FCS univariate method, on 28 datasets. By an initial assignment of a target pathway to each dataset, the authors evaluated the overall sensitivity and false positive rate produced when different ranking metrics were employed. In contrast to [40], the authors concluded that the ranking metric does have a significant impact on results and highlighted four metrics (i.e., moderated Welch test [42], minimum significant difference, absolute value of signal-to-noise ratio, and Baumgartner-Weiss-Schindler test [43]) that exhibited the best results with respect to overall sensitivity and false positive rate.

1. **Gene set – level statistics**

A number of gene set-level (i.e., global) statistics can be used for the calculation of the gene set score. This step is central to an enrichment analysis and ultimately determines whether a gene set is significantly enriched. In their comparative study, Ackermann and Strimmer [40] tested various gene-set level statistics, generally noting inconsistent performances across datasets (i.e., experimental and simulated). In their simulation, the authors found that the median of the transformed local statistic and the WRS test statistic can perform well when the competitive null hypothesis is tested and in the presence of outliers. Nonetheless, while the median was previously suggested in place of the mean, the authors note that the median, as well as the WRS test, may generate fewer overall results. A subsequent study by Hung *et al.* [44] also evaluated the impact of the global statistic on results by proposing a metric to assess the degree to which a gene set detected by a specific method can be reproduced by others. Here, the authors found that the WRS and Weighted Kolmogorov Smirnov (WKS) tests were able to obtain high scores on this metric and both are thus likely to be able to cover predictions by other evaluated global statistics.

1. **Applicability of various *omics* dataset types to enrichment analysis**

Given the steady shift from hybridization approaches to RNA sequencing, the applicability of enrichment methods, initially developed for microarrays, to RNA-seq data was specifically studied in Rahmatallah *et al.* [16]. The authors found that, with appropriate normalization, RNA-seq data is just as suitable as microarray data as input for these methods. It is worth noting that while the studies we have reviewed have largely been performed on real and/or simulated RNA-seq and microarray data, more recent studies have analyzed the performance of enrichment methods on other data types. For instance, a study by Holland *et al.* [45] ventures into an assessment of the applicability and performance of enrichment analysis on single cell RNA-seq data. Much in line with other benchmarks, they find that the gene sets selected are more sensitive to the analysis than the underlying statistic. Additionally, in Mora [46], the author also reviewed the use of methods for enrichment analysis besides RNA-seq and microarrays, including ChIP-Seq, SNP, methylation and non-coding RNA data, while Maksimovic *et al.* [47] benchmark the performance of enrichment analysis methods specifically designed for methylation data.

1. **Choice of background**

Enrichment methods which test the competitive null hypothesis, such as ORA, ​​assume that genes in a gene set are not differentially expressed with respect to genes not in the set (i.e., the background). The background can include genes in the rest of the experiment, genes from the gene set collection considered or even the entire genome. Geistlinger *et al.* [3] showcased how alternative approaches to select the background set (i.e., all genes in the experiment, all genes from the gene set collection considered, and a combination of these) in an over-representation analysis can lead to differences in the *p-*values of results of several orders of magnitude. Furthermore, In Wijesooriya *et al.* [48], the authors evaluated the suitability of the selection of background in nearly two hundred studies employing an ORA method and provided suggestions on the choice of background. They found that 95% of investigated studies failed to describe an appropriate background, with the majority of studies making no mention of the background selected and some using an inappropriate background, highlighting the broad oversight of this factor.

1. **Approaches that combine enrichment methods**

The Ensemble of Genes Set Enrichment Analyses (EGSEA) is an R package designed to combine the results of 12 distinct non topology-based methods by using different statistical methods to calculate a unified score for each gene set [49]. EGSEA has been implemented for RNA-seq data and leverages three gene set databases (i.e., MSigDB, KEGG, and GeneSetDB [50]). Similarly, an equivalent R package, EnrichmentBrowser, runs several topology and non-topology based methods in parallel, before results are combined and gene sets are given new rankings, based on a defined ranking and combination function [51]. Väremo *et al.* [52] provide the Piano R package which incorporates variations of the global statistic and can be used to conduct enrichment analyses for microarray and RNA-seq data through a variety of methods to obtain consensus results.

More recent developments include the decoupleR package [53], made available for similar purposes, yet expanding upon existing ensemble approaches through its adaptation to bulk, single-cell as well as spatial *omics* data, and the CPA (Consensus Pathway Analysis) web application, enabling non-bioinformatician users to conduct enrichment analyses on multiple methods and databases [54]. In the latter case, consensus pathway analyses can be conducted on eight disparate enrichment methods, using KEGG, GO or user uploaded gene set collections.

Finally, Ai and Kong [55] demonstrate an ML-based approach, Combined Gene set analysis incorporating Prioritization and Sensitivity (CGPS), to combine the *p-*values and ranks of the results of nine enrichment methods to train a support vector machine (SVM) that outputs a consensus score. The authors found that CGPS yielded a better performance than the two previously mentioned methods (i.e., EGSEA and EnrichmentBrowser) in identifying relevant gene sets.

1. **Multiple testing correction**

Another important methodological consideration includes the incorporation of corrections for multiple testing. A common scenario is one in which there are many gene sets for comparisons and few samples. As gene set collections can contain hundreds or even thousands of gene sets that are tested, type I errors can increase correspondingly to the number of tests [56]. While corrections are typically applied, depending on the dataset, enrichment method, and by extension, the model assumed by a given method, it may be advisable to ease or altogether forego multiple testing correction in certain cases, such as when a method is particularly conservative and is expected to yield little to no gene sets as significant [3].

# **References**

1. Ihnatova, I., Popovici, V., and Budinska, E. (2018). A critical comparison of topology-based pathway analysis methods. *PloS one*, *13*(1), e0191154. <https://doi.org/10.1371/journal.pone.0191154>
2. Bayerlová, M., Jung, K., Kramer, F., Klemm, F., Bleckmann, A., and Beißbarth, T. (2015). Comparative study on gene set and pathway topology-based enrichment methods. *BMC bioinformatics*, *16*(1), 334. <https://doi.org/10.1186/s12859-015-0751-5>
3. Geistlinger, L., Csaba, G., Santarelli, M., Ramos, M., Schiffer, L., Turaga, N., *et al.* (2020). Toward a gold standard for benchmarking gene set enrichment analysis. *Briefings in bioinformatics, 22*(1), 545-556. <https://doi.org/10.1093/bib/bbz158>
4. Maleki, F., Ovens, K. L., Hogan, D. J., Rezaei, E., Rosenberg, A. M., and Kusalik, A. J. (2019a). Measuring consistency among gene set analysis methods: A systematic study. *Journal of bioinformatics and computational biology, 17*(05), 1940010. <https://doi.org/10.1142/S0219720019400109>
5. Ritchie, M. E., Phipson, B., Wu, D. I., Hu, Y., Law, C. W., Shi, W., and Smyth, G. K. (2015). limma powers differential expression analyses for RNA-sequencing and microarray studies. *Nucleic Acids Research, 43*(7), e47-e47. <https://doi.org/10.1093/nar/gkv007>
6. Wu, D., Lim, E., Vaillant, F., Asselin-Labat, M. L., Visvader, J. E., and Smyth, G. K. (2010). ROAST: rotation gene set tests for complex microarray experiments. *Bioinformatics, 26*(17), 2176-2182. <https://doi.org/10.1093/bioinformatics/btq401>
7. Goeman, J. J., Van De Geer, S. A., De Kort, F., and Van Houwelingen, H. C. (2004). A global test for groups of genes: testing association with a clinical outcome. *Bioinformatics, 20*(1), 93-99. <https://doi.org/10.1093/bioinformatics/btg382>
8. Subramanian, A., Tamayo, P., Mootha, V. K., Mukherjee, S., Ebert, B. L., Gillette, M. A., *et al.* (2005). Gene set enrichment analysis: a knowledge-based approach for interpreting genome-wide expression profiles. *Proceedings of the National Academy of Sciences, 102*(43), 15545-15550. <https://doi.org/10.1073/pnas.0506580102>
9. Luo, W., Friedman, M. S., Shedden, K., Hankenson, K. D., & Woolf, P. J. (2009). GAGE: generally applicable gene set enrichment for pathway analysis. *BMC bioinformatics, 10*(1), 1-17. <https://doi.org/10.1186/1471-2105-10-161>
10. Tarca, A. L., Bhatti, G., and Romero, R. (2013). A comparison of gene set analysis methods in terms of sensitivity, prioritization and specificity. *PloS one, 8*(11), e79217. <https://doi.org/10.1371/journal.pone.0079217>
11. Ma, J., Shojaie, A., and Michailidis, G. (2019). A comparative study of topology-based pathway enrichment analysis methods. *BMC bioinformatics*, *20*(1), 1-14. <https://doi.org/10.1186/s12859-019-3146-1>
12. Ibrahim, M. A. H., Jassim, S., Cawthorne, M. A., and Langlands, K. (2012). A topology-based score for pathway enrichment. *Journal of Computational Biology, 19*(5), 563-573. <https://doi.org/10.1089/cmb.2011.0182>
13. Shojaie, A., and Michailidis, G. (2010). Network enrichment analysis in complex experiments. *Statistical applications in genetics and molecular biology, 9*(1). <https://doi.org/10.2202/1544-6115.1483>
14. Jacob, L., Neuvial, P., and Dudoit, S. (2012). More power via graph-structured tests for differential expression of gene networks. *The Annals of Applied Statistics, 6*(2), 561-600. <https://doi.org/10.1214/11-AOAS528>
15. Zyla, J., Marczyk, M., Domaszewska, T., Kaufmann, S. H., Polanska, J., and Weiner 3rd, J. (2019). Gene set enrichment for reproducible science: comparison of CERNO and eight other algorithms. *Bioinformatics*, *35*(24), 5146-5154. <https://doi.org/10.1093/bioinformatics/btz447>
16. Rahmatallah, Y., Emmert-Streib, F., and Glazko, G. (2016). Gene set analysis approaches for RNA-seq data: performance evaluation and application guideline. *Briefings in bioinformatics, 17*(3), 393-407. <https://doi.org/10.1093/bib/bbv069>
17. Michaud, J., Simpson, K. M., Escher, R., Buchet-Poyau, K., Beissbarth, T., Carmichael, C., *et al.* (2008). Integrative analysis of RUNX1 downstream pathways and target genes. *BMC genomics, 9*(1), 1-17. <https://doi.org/10.1186/1471-2164-9-363>
18. Tarca, A. L., Draghici, S., Bhatti, G., and Romero, R. (2012). Down-weighting overlapping genes improves gene set analysis. *BMC bioinformatics, 13*(1), 1-14. <https://doi.org/10.1186/1471-2105-13-136>
19. Tarca, A. L., Draghici, S., Khatri, P., Hassan, S. S., Mittal, P., Kim, J. S., *et al.* (2009). A novel signaling pathway impact analysis. *Bioinformatics, 25*(1), 75-82. <https://doi.org/10.1093/bioinformatics/btn577>
20. Gu, Z., Liu, J., Cao, K., Zhang, J., and Wang, J. (2012). Centrality-based pathway enrichment: a systematic approach for finding significant pathways dominated by key genes. *BMC systems biology, 6*(1), 1-13. <https://doi.org/10.1186/1752-0509-6-56>
21. Gu, Z., and Wang, J. (2013). CePa: an R package for finding significant pathways weighted by multiple network centralities. *Bioinformatics, 29*(5), 658-660. <https://doi.org/10.1093/bioinformatics/btt008>
22. Massa, M. S., Chiogna, M., and Romualdi, C. (2010). Gene set analysis exploiting the topology of a pathway. *BMC systems biology, 4*(1), 1-15. <https://doi.org/10.1186/1752-0509-4-121>
23. Martini, P., Sales, G., Massa, M. S., Chiogna, M., and Romualdi, C. (2013). Along signal paths: an empirical gene set approach exploiting pathway topology. *Nucleic acids research, 41*(1), e19-e19. <https://doi.org/10.1093/nar/gks866>
24. Gao, S., and Wang, X. (2007). TAPPA: topological analysis of pathway phenotype association. *Bioinformatics, 23*(22), 3100-3102. <https://doi.org/10.1093/bioinformatics/btm460>
25. Maleki, F., Ovens, K., McQuillan, I., and Kusalik, A. J. (2018). Sample size and reproducibility of gene set analysis. *IEEE International Conference on Bioinformatics and Biomedicine,* 122-129. <https://doi.org/10.1109/BIBM.2018.8621462>
26. Hänzelmann, S., Castelo, R., and Guinney, J. (2013). GSVA: gene set variation analysis for microarray and RNA-seq data. *BMC bioinformatics, 14*(1), 1-15. <https://doi.org/10.1186/1471-2105-14-7>
27. Tomfohr, J., Lu, J., and Kepler, T. B. (2005). Pathway level analysis of gene expression using singular value decomposition. *BMC bioinformatics, 6*(1), 1-11. <https://doi.org/10.1186/1471-2105-6-225>
28. Barbie, D. A., Tamayo, P., Boehm, J. S., Kim, S. Y., Moody, S. E., Dunn, I. F., *et al.* (2009). Systematic RNA interference reveals that oncogenic KRAS-driven cancers require TBK1. *Nature, 462*(7269), 108-112. <https://doi.org/10.1038/nature08460>
29. Grant, G. R., Manduchi, E., and Stoeckert Jr, C. J. (2007). Analysis and management of microarray gene expression data. *Current protocols in molecular biology, 77*(1), 19-6. <https://doi.org/10.1002/0471142727.mb1906s77>
30. Irizarry, R. A., Hobbs, B., Collin, F., Beazer‐Barclay, Y. D., Antonellis, K. J., Scherf, U., and Speed, T. P. (2003). Exploration, normalization, and summaries of high density oligonucleotide array probe level data. *Biostatistics, 4*(2), 249-264. <https://doi.org/10.1093/biostatistics/4.2.249>
31. Wu, Z., Irizarry, R. A., Gentleman, R., Martinez-Murillo, F., and Spencer, F. (2004). A model-based background adjustment for oligonucleotide expression arrays. *Journal of the American Statistical Association, 99*(468), 909-917. <https://doi.org/10.1198/016214504000000683>
32. Huber, W., Von Heydebreck, A., Sültmann, H., Poustka, A., and Vingron, M. (2002). Variance stabilization applied to microarray data calibration and to the quantification of differential expression. *Bioinformatics, 18*(suppl_1), S96-S104. <https://doi.org/10.1093/bioinformatics/18.suppl_1.S96>
33. Irizarry, R. A., Wu, Z., and Jaffee, H. A. (2006). Comparison of Affymetrix GeneChip expression measures. *Bioinformatics, 22*(7), 789-794. <https://doi.org/10.1093/bioinformatics/btk046>
34. Dillies, M. A., Rau, A., Aubert, J., Hennequet-Antier, C., Jeanmougin, M., Servant, N., et al. (2013). A comprehensive evaluation of normalization methods for Illumina high-throughput RNA sequencing data analysis. *Briefings in bioinformatics, 14*(6), 671-683. <https://doi.org/10.1093/bib/bbs046>
35. Anders, S., and Huber, W. (2010) Differential expression analysis for sequence count data. *Genome Biology 11*(10), R106. <https://doi.org/10.1186/gb-2010-11-10-r106>
36. Robinson, M. D., and Oshlack, A. (2010). A scaling normalization method for differential expression analysis of RNA-seq data. *Genome biology, 11*(3), 1-9. <https://doi.org/10.1186/gb-2010-11-3-r25>
37. Mortazavi, A., Williams, B. A., McCue, K., Schaeffer, L., and Wold, B. (2008). Mapping and quantifying mammalian transcriptomes by RNA-Seq. *Nature methods, 5*(7), 621-628. <https://doi.org/10.1038/nmeth.1226>
38. Young, M. D., Wakefield, M. J., Smyth, G. K., and Oshlack, A. (2010). Gene ontology analysis for RNA-seq: accounting for selection bias. *Genome biology, 11*(2), 1-12. <https://doi.org/10.1186/gb-2010-11-2-r14>
39. Maleki, F., Ovens, K., Hogan, D. J., and Kusalik, A. J. (2020). Gene set analysis: challenges, opportunities, and future research. *Frontiers in genetics, 11*(654). <https://doi.org/10.3389/fgene.2020.00654>
40. Ackermann, M., and Strimmer, K. (2009). A general modular framework for gene set enrichment analysis. *BMC bioinformatics, 10*(1), 1-20. <https://doi.org/10.1186/1471-2105-10-47>
41. Zyla, J., Marczyk, M., Weiner, J., and Polanska, J. (2017b). Ranking metrics in gene set enrichment analysis: do they matter?. *BMC bioinformatics, 18*(1), 1-12. <https://doi.org/10.1186/s12859-017-1674-0>
42. Welch, B. L. (1947). The generalization of ‘Student’s’ problem when several different population variances are involved. *Biometrika, 34*(1-2), 28-35. <https://doi.org/10.1093/biomet/34.1-2.28>
43. Baumgartner, W., Weiß, P., & Schindler, H. (1998). A nonparametric test for the general two-sample problem. *Biometrics,* 1129-1135. <https://doi.org/10.2307/2533862>
44. Hung, J. H., Yang, T. H., Hu, Z., Weng, Z., and DeLisi, C. (2012). Gene set enrichment analysis: performance evaluation and usage guidelines. *Briefings in bioinformatics, 13*(3), 281-291. <https://doi.org/10.1093/bib/bbr049>
45. Holland, C. H., Tanevski, J., Perales-Patón, J., Gleixner, J., Kumar, M. P., Mereu, E., et al. (2020). Robustness and applicability of transcription factor and pathway analysis tools on single-cell RNA-seq data. *Genome biology, 21*(1), 1-19. <https://doi.org/10.1186/s13059-020-1949-z>
46. Mora, A. (2020). Gene set analysis methods for the functional interpretation of non-mRNA data—Genomic range and ncRNA data. *Briefings in bioinformatics, 21*(5), 1495-1508. <https://doi.org/10.1093/bib/bbz090>
47. Maksimovic, J., Oshlack, A., and Phipson, B. (2021). Gene set enrichment analysis for genome-wide DNA methylation data. *Genome biology, 22*(1), 1-26. <https://doi.org/10.1186/s13059-021-02388-x>
48. Wijesooriya, K., Jadaan, S. A., Perera, K. L., Kaur, T., and Ziemann, M. (2022). Urgent need for consistent standards in functional enrichment analysis. *PLOS Computational Biology, 18*(3), e1009935. <https://doi.org/10.1371/journal.pcbi.1009935>
49. Alhamdoosh, M., Ng, M., Wilson, N. J., Sheridan, J. M., Huynh, H., Wilson, M. J., and Ritchie, M. E. (2017). Combining multiple tools outperforms individual methods in gene set enrichment analyses. *Bioinformatics*, *33*(3), 414-424. <https://doi.org/10.1093/bioinformatics/btw623>
50. Araki, H., Knapp, C., Tsai, P., and Print, C. (2012). GeneSetDB: a comprehensive meta-database, statistical and visualisation framework for gene set analysis. *FEBS open bio*, *2*, 76-82. <https://doi.org/10.1016/j.fob.2012.04.003>
51. Geistlinger, L., Csaba, G., and Zimmer, R. (2016). Bioconductor’s EnrichmentBrowser: seamless navigation through combined results of set-& network-based enrichment analysis. *BMC bioinformatics, 17*(1), 1-11. <https://doi.org/10.1186/s12859-016-0884-1>
52. Väremo, L., Nielsen, J., and Nookaew, I. (2013). Enriching the gene set analysis of genome-wide data by incorporating directionality of gene expression and combining statistical hypotheses and methods. *Nucleic acids research, 41*(8), 4378-4391. <https://doi.org/10.1093/nar/gkt111>
53. Badia-i-Mompel, P., Vélez, J., Braunger, J., Geiss, C., Dimitrov, D., Müller-Dott, S., *et al.* (2021). decoupleR: Ensemble of computational methods to infer biological activities from omics data. bioRxiv. <https://doi.org/10.1101/2021.11.04.467271>
54. Nguyen, H., Tran, D., Galazka, J. M., Costes, S. V., Beheshti, A., Petereit, J., *et al*. (2021). CPA: a web-based platform for consensus pathway analysis and interactive visualization. *Nucleic Acids Research*, gkab421,<https://doi.org/10.1093/nar/gkab421>
55. Ai, C., and Kong, L. (2018). CGPS: A machine learning-based approach integrating multiple gene set analysis tools for better prioritization of biologically relevant pathways. *Journal of genetics and genomics, 45*(9), 489-504. <https://doi.org/10.1016/j.jgg.2018.08.002>
56. Korthauer, K., Kimes, P. K., Duvallet, C., Reyes, A., Subramanian, A., Teng, M., *et al.* (2019). A practical guide to methods controlling false discoveries in computational biology. *Genome biology, 20*(1), 1-21. <https://doi.org/10.1186/s13059-019-1716-1>
